# Influence of oceanographic structures on foraging strategies: Macaroni penguins at Crozet Islands

**DOI:** 10.1186/s40462-015-0057-2

**Published:** 2015-09-21

**Authors:** Cecile Bon, Alice Della Penna, Francesco d’Ovidio, John Y.P. Arnould, Timothée Poupart, Charles-André Bost

**Affiliations:** Centre d’Etudes Biologiques de Chizé, UMR 7372, CNRS - Université de La Rochelle, 79360 Villiers en Bois, France; Sorbonne Universités, UPMC Université Paris 06, UMR 7159, LOCEAN-IPSL, F-75005, Paris, France/Université Paris-Diderot/CSIRO-UTAS Quantitative Marine Science Program, IMAS, Private Bag 129, Hobart, TAS 7001 Australia; Sorbonne Universités, UPMC Université Paris 06, UMR 7159, LOCEAN-IPSL, F-75005 Paris, France; School of Life and Environmental Sciences, Faculty of Science, Engineering and the Built Environment, Deakin University, 221 Burwood Highway, Burwood, VIC 3125 Australia

## Abstract

**Background:**

In the open ocean, eddies and associated structures (fronts, filaments) have strong influences on the foraging activities of top-predators through the enhancement and the distribution of marine productivity, zooplankton and fish communities. Investigating how central place foragers, such as penguins, find and use these physical structures is crucial to better understanding their at-sea distribution. In the present study, we compared the travel heading and speed of the world’s most abundant penguin, the Macaroni penguin (*Eudyptes chrysolophus*), with the distribution of surface physical structures (large-scale fronts, eddies and filaments).

**Results:**

The study was performed during December 2012 in the Crozet Archipelago (46.42° S; 51.86° E), South Indian Ocean. Six males at incubation stage were equipped with GPS loggers to get their trajectories. We used Eulerian and Lagrangian methods to locate large-scale fronts, mesoscale eddies (10–100 km) and part of the sub-mesoscale structures (<10 km, filaments) at the surface of the ocean. By comparing the positions of birds and these structures, we show that Macaroni penguins: i) target the sub Antarctic Front; ii) increase their foraging activity within a highly dynamic area, composed of eddy fields and filamentary structures; and iii) travel in the same direction as the predominant currents.

**Conclusions:**

We show that penguins adjust their travel speed and movement during their whole trips in relation with the oceanographic structures visited. At a large scale, we hypothesize that Macaroni penguins target the sub Antarctic Front to find profitable patches of their main prey. At finer scale, Macaroni penguin may adopt a horizontal drifting behavior in strong currents, which could be a way to minimize costs of displacement.

## Background

In the open ocean, the distribution and abundance of marine organisms is related to physical processes at different spatial and temporal scales [1]. Many studies have provided evidence of strong relationships between the foraging movements of top-predators and the distribution of mesoscale (10–100 km), predictable oceanographic structures such as large fronts and eddies (e.g. [2–4]). Recently, the relationships between marine top-predators and sub-mesoscale (<10 km) features (e.g. filamentary structures) have also received growing interest and have triggered the development of new Eulerian (observations at a given time, in the “non-moving” frame of reference of the bathymetry) and Lagrangian diagnostics (from the frame of reference of flowing water particle). Lagrangian diagnostics enable the analysis of the temporal and spatial variability of oceanographic features to identify physical structures like eddies, fronts, and part of the filament variability. Such structures have been shown to affect the distribution and growth of phytoplankton because their lateral and vertical transport properties influence the supply and retention of nutrients in the euphotic layer from deeper waters [5, 6]. Correspondingly, such aggregations of primary production can influence food web dynamics due to their profitability for all species from grazers to top predators [1, 7, 8]. Indeed, it has been shown that several top predators use eddies (e.g. [9, 10]), currents and associated filaments to forage (e.g. [8, 11]).

Relatively, few studies have focused on penguins [10, 11] despite their key role in marine food webs [12]. These non-flying, diving predators are highly constrained in their foraging range because of their low travelling speed and high cost of transport. It might be expected, therefore, that oceanic penguins should target sub-meso and mesocale structures during their at-sea activities to maximise their foraging efficiency [10].

Consequently, we investigated the at-sea foraging movements of the Macaroni penguin (*Eudyptes chrysolophus*) in a highly dynamic marine environment: the waters around the Crozet Archipelago in the South Indian Ocean. The Macaroni penguin is a pelagic predator, diving within the mixed layer to mean depths of 50 m (up to 163 m, [13]) to capture crustaceans and myctophid fish [14–16]. The species exhibits large flexibility in its foraging range, exploiting frontal structures or the shelf area according to the breeding requirements [17, 18]. While the world population is currently decreasing [19, 20], it is still the most abundant penguin species and the largest marine biomass consumer among seabirds [12]. The Crozet Archipelago is a breeding stronghold for the species [19].

Our aim was to identify how Macaroni penguins use oceanic structures to forage at different spatial scales, from large-scale (front) to meso- (eddies) and sub-mesoscale filamental structures [21]. We attempted to answer the following questions: i) do Macaroni penguins adjust their spatial movements with the regional circulation of currents?; and ii) how do they adjust their foraging behavior within meso- and sub-mesoscale structures? We address these questions by investigating the relationships between the spatial behavior of penguins and: i) the presence of persistent, large-scale frontal structures; ii) the occurrence of eddies and filamentary structures; and iii) the adjustment of their travel speed with the encountered currents. We hypothesize that penguins would target these structures, reducing travel speed within eddies and filamentary structures to foraging intensively, as such behaviors should be advantageous with respect to travel costs.

## Results

**Fig. 1:**
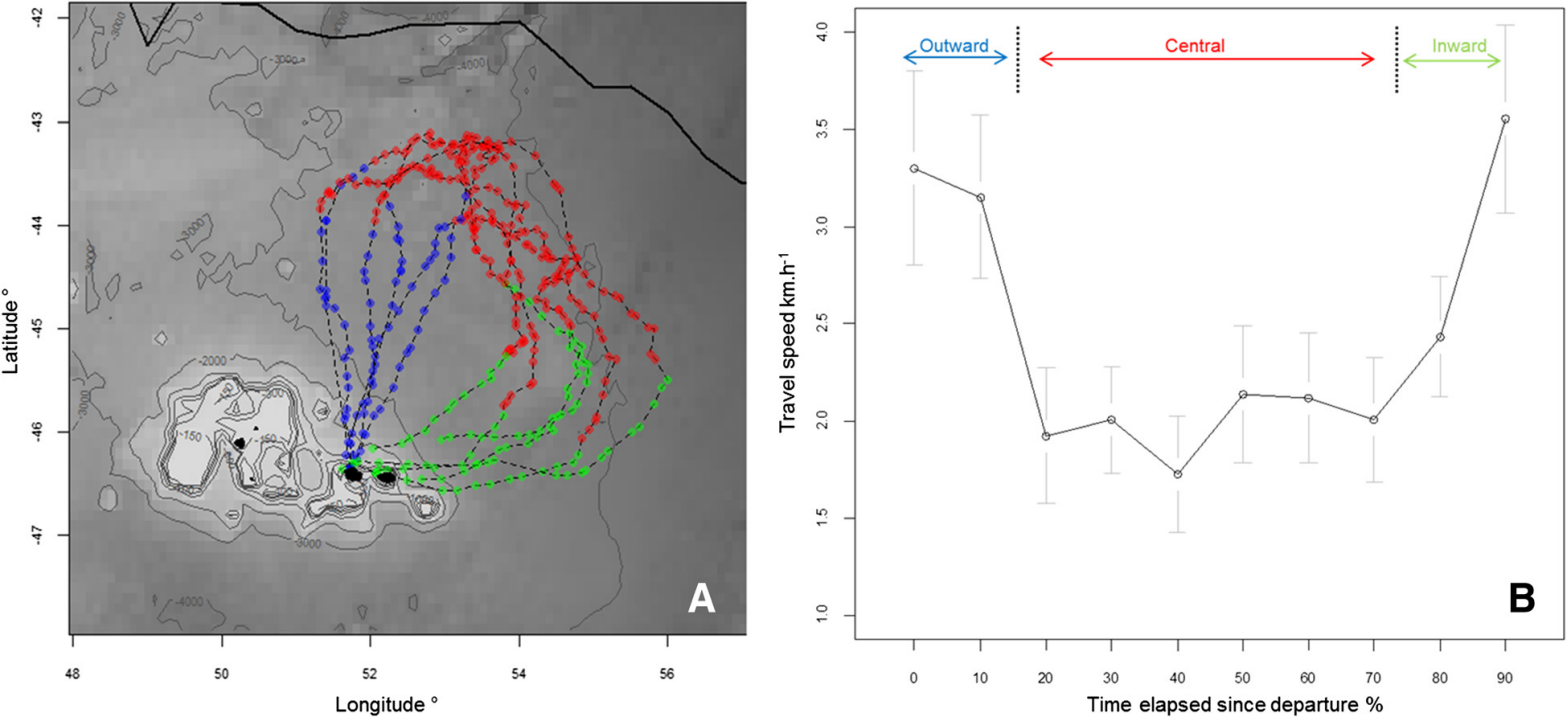
Trips and travel speed of six incubating macaroni penguins presented on a bathymetry map around the Crozet islands. **a** The three phases defined by the variation in heading velocity are represented in distinct colours: Outward: *blue*, Central: *red*, Inward: *green. Black line*: sub Antarctic Front. **b** The travel speed was averaged for each 10 % of time elapsed since the departure of travel. *Arrows* indicate the separation of the trip in three phases

After their foraging trips (18 ± 2 days), all the instrumented penguins were re-captured upon returning to their colony having increased their body mass (subsequently, all pairs successfully fledged their chicks). Data from one GPS were lost due to technical failure and thus six tracks were analyzed in the present study. Individuals performed long clockwise looping trips, heading north towards the SAF, up to 388 km in a region encompassing positive and negative eddies, before returning to the colony (Fig. 1a). The central phase of their trips were longer (435.7 ± 69.9 km; 9.66 ± 1.35 d) than the outward (280.8 ± 38.9 km; 3.80 ± 0.47 d) and inward phases (237.6 ± 72.8 km; 9.66 ± 1.35 d, Kruskal-Wallis test on duration: *X*^*2*^ = 11.94, df = 2, *p* < 0.01, Table 1).

**Table 1:** Main characteristics of foraging trips of six Macaroni penguins

Trip phase	Duration (j)	Travel speed (km.h ^−1^)	Heading velocity (km.h ^−1^)	Current speed (km h ^−1^)	Animal direction °	Current direction °
Outward	3.8 ± 0.5	3.6 ± 1.3	3.5 ± 1.3	0.2 ± 0.2	11.4 ± 22.4	61.4 ± 97.8
*n = 86*						
Central	9.7 ± 1.4	1.9 ± 1.1	1.7 ± 0.9	0.9 ± 0.4	141.4 ± 68.8	118.1 ± 37.9
*n = 233*						
Inward	3.8 ± 1.1	3.3 ± 1.3	3.2 ± 1.3	0.3 ± 0.1	233.2 ± 45.0	246.3 ± 101.0
*n = 96*						

*n* number of gps localisations

The distance travelled every 6 h was on average 15.2 ± 12.7 km. The travel speed was significantly lower within the central phase (outward: 3.57 ± 1.25 km · h ^−1^, central: 1.93 ± 1.05 km · h ^−1^, inward: 3.30 ± 1.30 km · h ^−1^, Kruskal-Wallis test: *X*^*2*^ = 103.97, df = 2, *p* < 0.0001, Table 1, Fig. 1b).

**Fig. 2:**
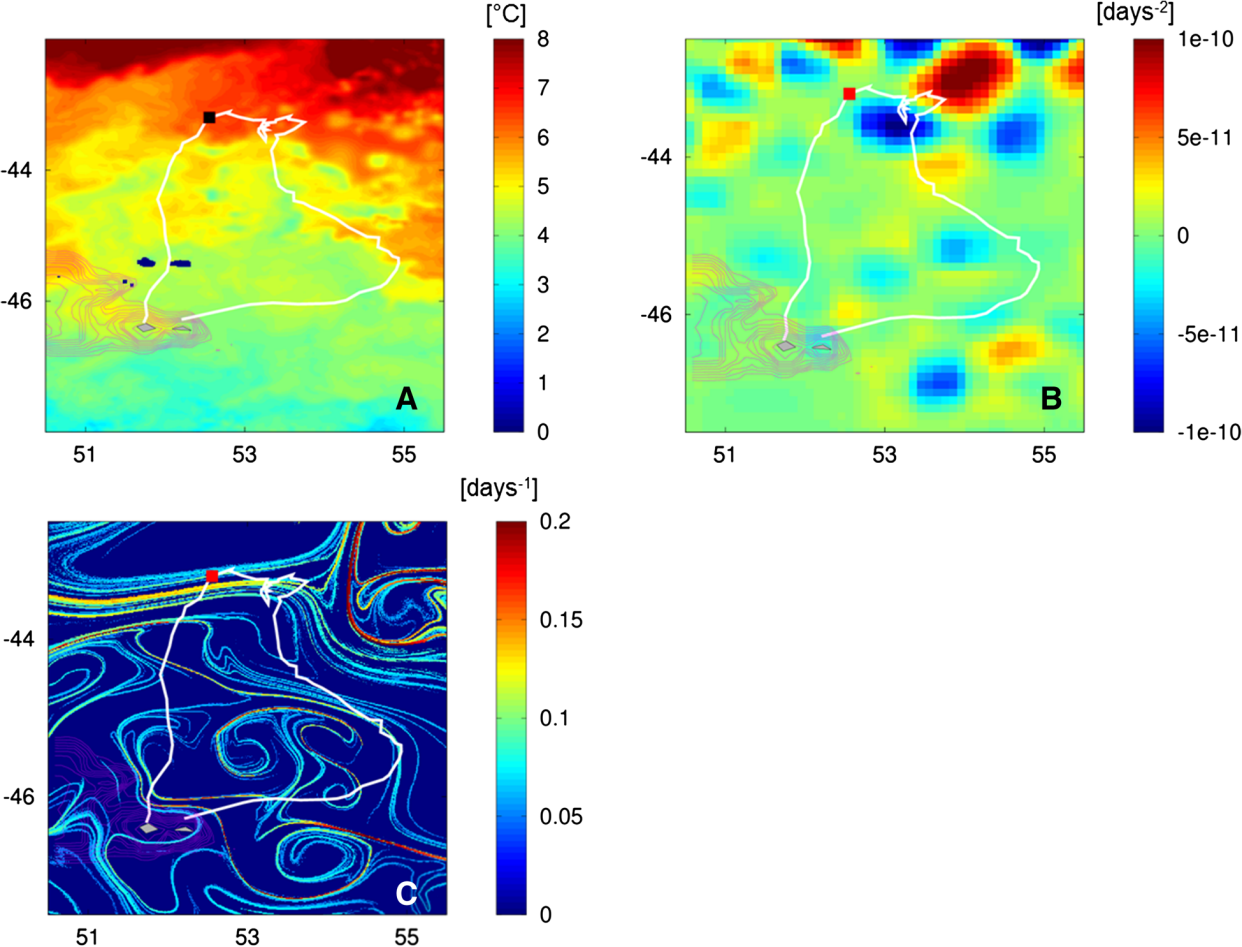
The positions of one bird overlaid on oceanographic features. **a** Map of Sea Surface Temperature (°C). **b** Okubo–Weiss parameter: eddy cores are characterized by negative values. **c** Finite-size Lyapunov exponents (δ _0_ = 0.01°, δ_f_ = 0.6°): larger values indicate stronger transport barriers

There was a gradient in SST encountered by penguins during their trip from ~4 °C at the colony to 8 °C at the lowest latitudes visited (~43° S, Fig. 2a). The SST was highly positively correlated with the current speed. This suggests that the warmer waters encountered by penguins located at the lowest latitudes were also in the strongest currents visited (Spearman correlation test: 7068809, R^2^ = 0.40, *p* < 0.001).

### Penguin and mesoscale eddies

During the central phase of their trips, Macaroni penguins foraged at the edge of two large eddies, situated in the vicinity of the SAF (Fig. 2b). These two eddies were located to the south of a large eddy field which was not used by the birds. Overall, 63 % of the locations associated with an eddy were within the central phase whereas 11 and 26 % were within the outward and inward phases, respectively (Table 2). This indicates that the main eddy activity was observed within the central phase where the penguins had reduced swimming speed. Indeed, the degree of association with eddies was 37 % in the central phase, 17 % in the outward phase and 38 % in the inward phase (Table 2).

**Table 2:** Distribution of eddies and filaments within trips of six macaroni penguins

		Eddies		Filaments	
Trip Phase	n_*locs averaged on indiviudals*_	Distribution of eddy locations	Degree of association within phase	Distribution of filaments	Degree of association within phase
		%–n_*locs*_	%–n_*locs*_	%–n_*locs*_	%–n_*locs*_
Outward	14 ± 4	11 %–15	17 %–3 ± 1	20.8 %–22	21.7 %–3.0 ± 1.0
Central	38 ± 5	63 %–87	37 %–15 ± 6	66.7 %–61	27.6 %–9.6 ± 6.0
Inward	16 ± 4	26 %–36	38 %–6 ± 3	12.5 %–10	12.5 %–2.0 ± 1.0

*n*_*locs*_ number of locations. *Mean ± SD* Number of locations within eddies/filaments averaged within each phase for each individual

**Fig. 3:**
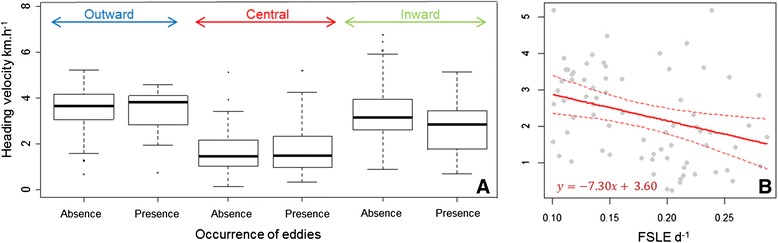
Heading velocities related to eddies and filament characteristics. **a** Distribution of heading velocity inside or outside of eddies within each trip phase. *Arrows* indicate trip phases. **b** Heading velocity in relation to the FSLE values within filaments (FSLE >0.1d ^−1^). *Red line* is the regression line resulting from the M4 model. *Dashed lines* indicate 95 % confidence intervals of predictions

Examination of time spent within eddies (successive locations in an eddy) indicates penguins spent more time within eddies in the central phase (43 ± 25 h, 11 cases) than in the transit phases (25 ± 10 h, 10 cases, Mann-Withney: *U* =81.5, *P* = 0.066). The retention parameter was small for eddies in the central phase (9.7 ± 15 d, *n =* 60 locations) since 80 % of water parcels had been recirculating within the eddy for less than 8 d. In transit phases, the retention parameter of eddies was significantly higher (16 ± 15 d, *n =* 32 locations, Mann-Withney test: *U* =658, *P* = 0.013). Finally, the three mixed models built for each phase indicated that penguins significantly slowed down when they were inside eddies in the inward phase, contrary to that observed in the two other phases (Table 3 models M1, M2,M3, Fig. 3a).

**Table 3:** Influence of the occurrence of eddies, filaments and current speed on heading velocity

HV ~ OW _category_	Intercept	Presence of eddy	*P*_*intercept*_ */P*_*variable*_	_∆*AIC* Null_
M1 – Outward phase	**3.57 ± 0.20**	−0.14 ± 0.40	<0.0001/NS	+1.87
M2 – Central phase	**1.62 ± 0.11**	0.12 ± 0.16	<0.0001/NS	+3.30
M3 – Inward phase	**3.43 ± 0.25**	**−0.635 ± 0.29**	<0.0001/< 0.05	- 2.14
HV ~ FSLE _(>0.1)_	Intercept	FSLE _(>0.1)_	*P*_*intercept*_ */P*_*variable*_	_∆*AIC* Null_
M4 **–** Whole trip	**3.60 ± 0.51**	**−7.29 ± 2.78**	<0.001/< 0.05	- 6.40
TV ~ Current speed	Intercept	Current speed	*P*_*intercept*_ */P*_*variable*_	_∆*AIC* Null_
M5 **–** Whole trip	**3.51 ± 0.19**	**−1.51 ± 0.24**	<0.001/<0.001	−27.38

Linear mixed models were independently built with individual bird included as a random effect (*n* = 6) for each explanatory variable. Response variables are heading velocity (HV) and travel velocity (TV). The Okubo-Weiss parameter is a binary factor coding for the occurrence of eddies (0: absence, 1: presence). Current speed and FSLE are continuous variables. Only FSLE values >0.1d^−1^ were selected to test for the influence of filaments on HV when penguins were within a filament. Significant coefficients (mean ± se) are in bold. *P p*.value, *NS* non-significant. _∆*AIC* Null_ shows the AIC deviation from AIC of the null model

### Penguin movements and filaments

Filaments identified by the FSLE method were present over the whole area prospected by the penguins (Fig. 2c). At the trip scale, we observed high inter-individual variation in the level of association with filaments (n_locs within filaments_/n_locs total)_ (from 5.6 to 35.9 %). Across all trips, 66.7 % of the locations associated with filaments were located within the central phase of the foraging trip where penguins reduced travel speed (20.8 and 12.5 % in outward and inward phases, respectively, Table 2). The degree of association was significantly higher in the central phase since 27.6 % of locations were within filaments. In the two others phases, the number of locations within filaments were significantly lower (Kruskal-Wallis test: *X*^*2*^ = 6.976, df = 2, *p* < 0.05, Table 2). FSLE values of the filaments were significantly higher at the central phase (0.20 ± 0.05 d ^−1^) than at the outward (0.15 ± 0.04 d ^−1^) and inward (0.12 ± 0.02 d ^−1^) phases (Kruskal-Wallis test: *X*^*2*^ = 18.603, df = 2, *p* < 0.001). Once individuals were inside the filaments, they slowed down more when FSLE values were higher (Table 3 model M4, Fig. 3b).

### Penguin movements and currents

**Fig. 4:**
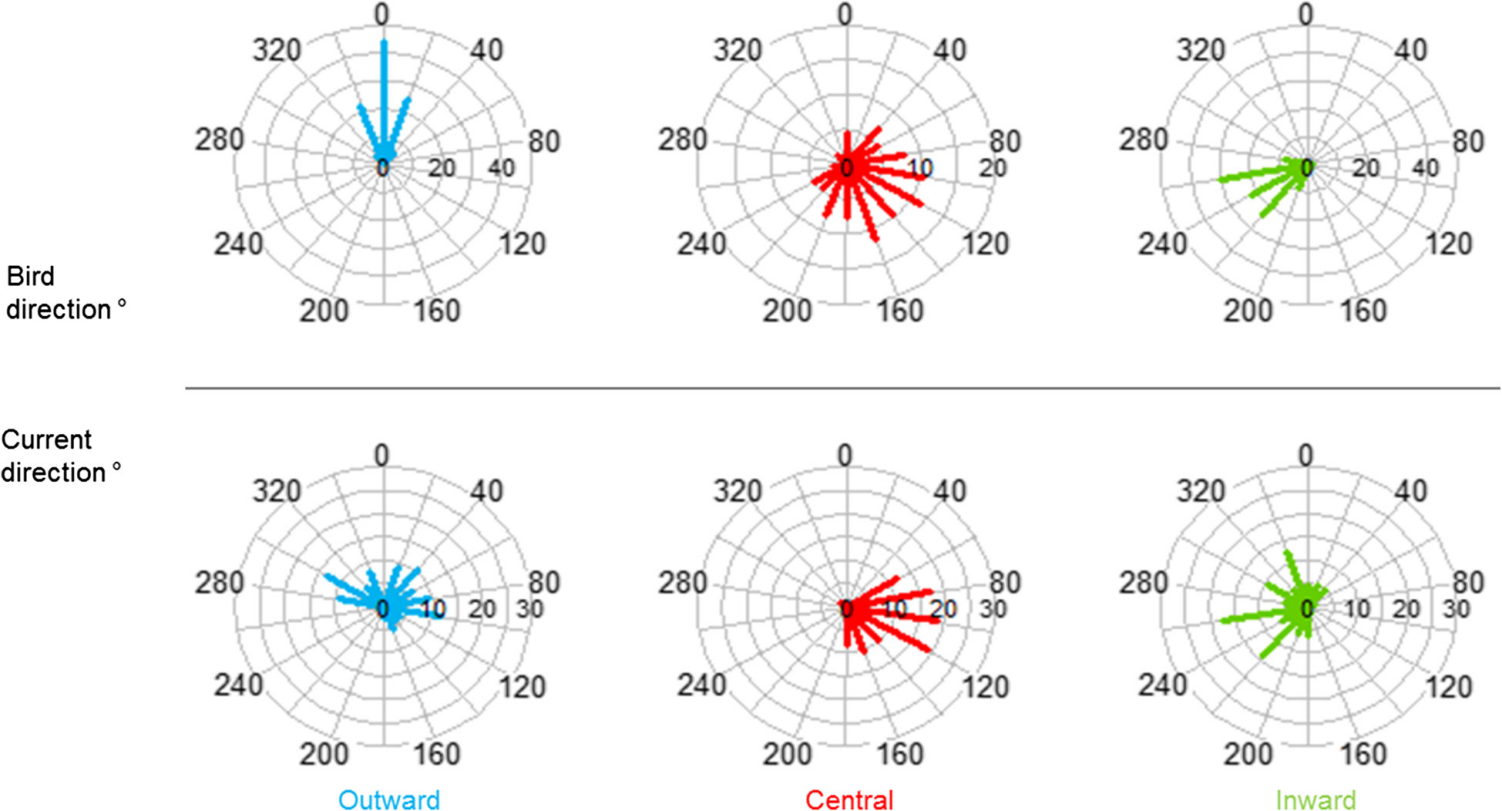
Angular deviations in the headings of penguins and ocean currents within each phase of trips. The proportion (%) of deviations between the direction of travel of ocean currents and the tracked penguins, computed at the resolution of 20 °

At the whole-trip scale, at-sea movements of individuals seem to be strongly modified by the currents encountered. Firstly, travel speed was negatively correlated with the current speed indicating that penguins decelerated when they encountered stronger currents (Table 3 model M5). Indeed, during the outward phase, the current speed was generally low and no clear relationship was observed between the penguins and direction of the current (Table 1, Fig. 4).

In the central phase, penguins shifted toward a south-eastern direction (141.40 ± 68.78°) with a travelling speed significantly lower than during the two other trip phases (Kruskal-Wallis test: *X*^*2*^ = 103.9734, df = 2, *p* < 0.001). At that time the currents were significantly faster than during the two other phases (Kruskal-Wallis test: *X*^*2*^ = 169.90, df = 2, *p* < 0.001), up to 1.6 km · h ^−1^, and mainly oriented in the same direction as the penguins’ headings (118.10 ± 37.86°, Fig. 4). A strong correlation between the directions of the penguins and currents were also found (circular Pearson test: 5. 78, R^2^ = 0.40, *p* < 0.001). A substantial proportion (25.3 %) of heading velocities was <1 km · h ^−1^ indicating displacement close to that of the current speed suggesting a possible drifting behavior by the birds.

During the inward phase, the penguins moved quickly back to the colony and their paths were mostly orientated south-westerly (Fig. 4). The weak currents were also oriented south-westerly and positively correlated with the penguins’ main direction (circular Pearson test: 4.55, R^2^ = 0.50, *p* < 0.001).

## Discussion

The salient findings of this study can be summarized as follows. Firstly, tracked Macaroni penguins performed long looping trips north of Crozet towards a predictable large-scale frontal structure, the SAF. The similarity in their swimming direction strongly suggests a common use of oceanographic features. Secondly, during the central phase of their trip, the penguins slowed down and foraged inside large eddies, following a northeast flow. Overall, in accordance with our assumptions, the penguins adjusted their travel speed and movement throughout their trips in relation to the oceanographic structures visited.

### Use of large-scale circulation around the Crozet Archipelago

The foraging movements of Macaroni penguins toward the SAF demonstrate these diving predators use predictable, large-scale physical feature in agreement with our first assumption. This is consistent with the highest mean seabird species richness and diversity in the South Indian Ocean having been reported at the SAF [22]. This diversity and abundance is driven by the high concentration of chlorophyll-*a* and macro-zooplankton within the SAF, resulting from increased water column stability and availability of nutrients [23].

Recent *in situ* oceanographic sampling and remote sensing data [24] have shown that a predictable phytoplankton bloom occurs north of Crozet [25] each year in early September. North of Crozet, the SAF deviation creates a closed area with long residence time which allows dissolved iron from land or sediments of the Crozet plateau to fertilize the water during winter. These conditions enhance the development of the phytoplankton bloom [26, 27] which reaches a peak in late October i.e. during the period before the at-sea sojourns of incubating males tracked in our study. During this time, Macaroni penguins mainly feed on euphausiids, (primarily *Euphausia valentini* and *Thysanoessa macrura*), amphipods (*Themisto gaudichaudii)* and myctophid fish (*Krefftichthys anderssoni spp.*) [15, 16], which have been found in high concentrations within the PFZ [28-30].

### Foraging behavior in meso- and sub-mesoscale structures

At a fine scale, individuals modified their swimming behavior when entering meso- (eddies) and sub-mesoscale (filaments) structures. In agreement with other diving predators [9, 10, 31], Macaroni penguins slowed down, suggesting they undertook more intensive foraging activity, during this phase characterized by an important eddy field. The greater relative abundance of young eddies in this phase compared to the two other phases confirms that the central phase is located in a branch of the SAF characterized by an important mixing activity [32]. Numerous studies have shown that several trophic levels of organism can aggregated within eddies [33, 34] and, through a cascading effect, many predators could benefit from this [10, 31, 35]. In addition, in this study, penguins showed no difference in heading velocity within and outside of eddies in the central phase, whereas currents were stronger and filamentary activity higher than in the other phases. We suggest that the prey field was extended at the spatial scale of the branch of the SAF and this hypothesis is coherent with the spatial structure of the annual phytoplankton bloom [36]. While it is reasonable to assume that local variations of prey density exist at finer scale, at the sub-mesoscale, the sampled distance between locations (tens of km) was too large to detect variations in heading velocity responding to such prey distributions.

During outward and inward phases, penguins did not respond in the same way to the presence of eddies. Eddies were not visited in the outward phase since no changes of heading velocity were observed. However, a significant slow-down was shown in the inward phase within an eddy. As suggested by Cotté et al*.* [31], all eddies are not used and it would depend on their life-time and history. In our study, eddies in transit phases presented a retention time significantly higher than in the central phase. As eddy cores present a relatively poorly mixing environment [37], they retain nutrients and thus probably enhance biological productivity and prey aggregation. The weak currents inside the eddy cores may also explain the reduced travelling speed of individuals as they foraged inside these structures. Thus, the behavioral changes observed in the eddy during the inward phase could indicate that the eddy is profitable.

Concerning the sub-mesoscale activity, the central phase was also the area where the filamentary structure was the highest, confirming the dynamic character of the area. This is to be expected as filaments are mostly formed from eddy-eddy interactions [38]. Furthermore, once individuals were inside filaments, they slowed down more as the horizontal stirring increased. This is consistent with the trapping characteristics of these structures retaining chlorophyll and thus attracting species in the upper trophic levels [6, 39]. However, no difference was detected in swimming behavior inside and outside the filaments, in contrast to that observed with eddies. This may be due to several factors.

Firstly, crustaceans and fish are mobile in comparison to the phytoplankton patches which are transported by currents, which could induce a more dispersed spatial distribution outside the filaments. Secondly, these transport barriers are mostly located at eddy edges [37]. Thus, Macaroni penguins may have responded to the productivity associated with eddy characteristics and not to the filament properties (i.e. at a finer scale). Finally, any adjustment of movements by penguins to filament characteristics may not have been detected due to the spatial resolution of the datasets used (i.e. altimetry data at 0.33 ° and 1 week, GPS locations limited to 6 h intervals, tens of km).

### Currents

Throughout the different phases of their foraging trips, Macaroni penguins exhibited marked shifts in their travel speed in relation to the current directions encountered. The heading velocities (HV) were generally much greater than the fastest encountered currents (>0.8 km · h ^−1^). However, in areas where currents were fastest, 30 % of trip segments were associated with an HV of less than 1 km · h ^−1^. This indicates a travel speed close to the current speed which strongly suggests individuals were drifting horizontally.

In marine predators, surface drift behaviors have been explained as a consequence of different processes. Firstly, current speeds may be similar to the swimming ability of the studied species. This results from the current’s influence on the animals’ trajectories [40]. Secondly, drift behavior could occur at night in daytime foragers resting during multi-day trips [17]. Thirdly, horizontal drift behaviors could be indicative of an increase in vertical foraging activity.

Finally, the maximum swimming speed of Macaroni penguins (up to 10 km · h ^−1^ [41]) is high compared to the current speed. Hence, the low HV observed at the central phase of the foraging trips in the present study could correspond to an increase in diving activity resulting in passive horizontal movement (drift).

Association with the local currents could be a good way to minimize transports costs. Indeed, from the start of the breeding cycle until the creching phase, males have to endure two extended fasting periods. The first lasts ~ 35 d (i.e. from the arrival of the birds at the colony until their departure after the first long incubation period) and the second occurs at the end of their first post-incubating trip until the end of brooding (i.e. ~35 days [42, 43]). Thus, during their first post-incubation trip, males are highly energetically constrained as they have to restore their body condition and acquire enough reserves to prepare for the next fasting event. Consequently, individuals would gain significant energetic advantages by adopting behaviors that avoided swimming against currents. Our results support this hypothesis. Such behavior has been observed in other oceanic penguins (e.g. king penguins *Aptenodytes patagonicus*, Magellanic penguins *Spheniscus magellanicus*) at a time when they also need to quickly progress to favorable foraging areas [10, 44].

## Conclusions

This work confirms the high dependence of Macaroni penguins on large-scale frontal zones such as the SAF in the Crozet area, a key breeding area for the species. This is the first demonstration of such strong dependence to the SAF for the Crozet Macaroni population. In addition, our study highlighted the role of currents and eddy activity on the foraging behavior of a diving predator. In future studies, the adjustment of movement behavior to filaments should be tackled at a finer scale with a more precise overlap between predator movements and the location of frontal structures. Investigating diving success in these structures would be also of special interest. Furthermore, analysis of whether the drift behavior is actually associated with more intensive foraging should be undertaken, potentially using 3D movement data. Finally, it would also be important to know if such behavior is exhibited during other periods where penguins are subjected to other major energetic constraints such the creche phase or pre-moulting period [45].

## Methods

**Fig. 5:**
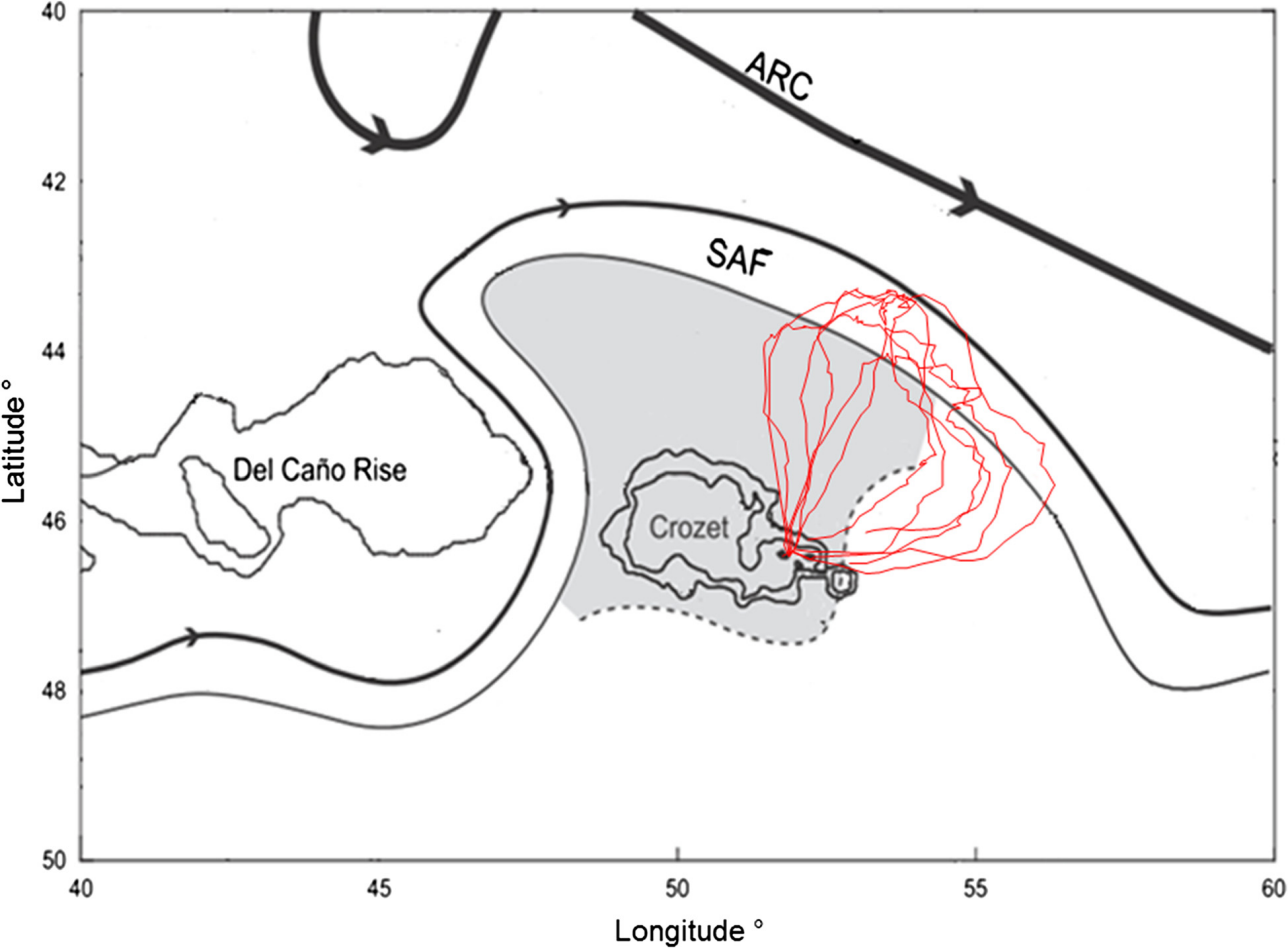
Map of oceanographic fronts taken from Pollard and Read (2001, [46]). Macaroni trips (*red*) are shown in red. SAF: sub Antarctic Front. ARC: Agulhas Return Current

The study was carried out at the Jardin Japonais colony, Possession Island (46°21′ S, 51°43′ E), Crozet Archipelago (hereafter, referred to as Crozet). The archipelago lies on the Crozet Plateau (45–47° S, 49–51° E) (150 km of width, less than 500 m deep) and at the northern extent of the eastward flowing Antarctic Circumpolar Current (ACC) [36]. The Crozet Plateau deflects one of the current’s major branches, the sub Antarctic Front (SAF), to the south of the Del Caño Rise before flowing northward under the influence of the local bathymetry. North of the plateau, it turns eastward under the influence of the Agulhas Return Current and the SubTropical Front [36]. The SAF is associated with strong eastward currents, located between 42 and 43° S, whereas a weak circulation dominates between the Crozet shelf and 44° S [25] (Fig. 5, [46]).

During the 2012 Austral summer, a total of 7 adult breeding males (incubation stage) were captured (20–21 November) before the departure for their first long trip. The penguins were instrumented with a GPS logger (Fastloc 2, Sirtrack, Havelock North, N.Z.) when leaving their colony. The devices were programmed to record location every 15 min. Each logger was attached to the lower dorsal feathers along the central mid-line, to minimize drag effects [47], with instantaneous cyano-acrylate glue (Loctite 401 Prism, Instant Adhesive, Hempstead, Hertfordshire, HP2 4RQ UK) and waterproof tape (Tesa 4651, Tesa Tape, Quickborn str 24, Hamburg 20253, Germany), and further secured by two cables ties. The duration of the instrumentation procedure lasted <15 min. All the birds were recaptured upon their return to the colony and the equipment removed.

### Oceanographic data

Altimetry maps were obtained from the CNES/CLS AVISO website [48] with spatial and temporal resolutions of 0.33° and 1 week, respectively [49]. Altimetry was used to compute the velocity of horizontal currents and to identify sub- and mesoscale physical structures. The currents’ velocities were compared with the velocity of penguins, called travelling velocity (TV), determined from GPS tracking, by computing the heading velocity (HV) [50], which is defined as:*v*_(*heading*)_ = *v*_(*tracking*)_ − *v*_(*currents*)_.

To identify sub- and mesoscale structures, we used Eulerian and Lagrangian diagnostics: the Okubo-Weiss (OW) parameter to identify eddies, the Finite Size Lyapunov Exponent (FSLE) to identify filaments, and the Retention Parameter (RP) to quantify for how long the water parcels within an eddy have been recirculating:

The Okubo-Weiss parameter OW [37, 51] is defined as:

$$W=s{n}^2+s{s}^2-{\omega}^2$$

where *sn* and *ss* are the normal and shear components of strain and ω is the relative vorticity of the flow. The sign of this parameter locates eddies as regions with negative OW parameters (vorticity is dominant) and background as oceanic regions of small negative and positive OW parameters (strain is dominant, absence of eddies). Following Bailleul et al*.* [9], we used the *W*_*o*_ = 0.2*σ*_*w*_ (*σ*_*w*_ is the standard deviation of *W* in the whole domain) threshold to separate vorticity-dominated (*W* < − *W*_*O*_, presence of eddy) regions from strain-dominated regions (*W* ≥ *W*_*O*_, absence of eddy) and the background field (|*W*| ≤ *W*_*O*_).

The Finite Size Lyapunov Exponents (FSLE) method provides a direct measure of the amount of local stirring by mesoscale currents. It can be used to identify transport barriers along which water parcels are stretched into elongated structures (hereafter, termed filaments), typically in the region between eddies [38]. The FSLE computes the backward-in-time divergence (i.e. convergence) of particles initially in close proximity to each other and is commonly used as an indicator of frontal activity and stirring intensity [52].

It is computed as:

$$\lambda \left(x,t,{\delta}_0,{\delta}_f\right)=\frac{1}{\tau } log\frac{\delta_f}{\delta_0}$$

Where *δ*_0_ represents the initial separation of water parcels, and *τ* the time taken for the water parcels to reach a separation *δ*_*f*_ . For the present study, the parameters used for the calculation were *δ*_*f*_ = 0.6 degrees, *δ*_0_ = 0.01 degrees and *τ* had a maximum limit of 100 days.

Highest FSLE values are associated to formerly distant water masses, whose confluence creates a transport front [52]. Here, we used FSLE >0.1 d^−1^ as indicators of the presence of a transport front. FLSE ridges can represent the edges of mesoscale eddies but also the convoluted boundaries of sub-mesoscale filaments.

The Retention Parameter (RP) computes the backward trajectories of simulated water parcels from negative OW regions (i.e. eddies) and measures for how long each water parcel has been within the same OW negative patch. This quantity corresponds to the time the water has been recirculating within the eddy [37].

The SAF was identified as the 8 °C sea surface isotherm during the period corresponding to the measured trajectories (22/11/2012-11/12/2012) [53, 54]. Sea Surface Temperature (SST) was obtained from the G1SST (Global 1-km Sea Surface Temperature) Level 4 product from GHRSST (Group for High Resolution Sea Surface Temperature [55]). In addition, to provide context for primary production in the regions explored by the tracked penguins, we used sea-surface chlorophyll-*a* concentration data from GlobColour [56] with a daily average resolution of 9 km^2^.

### Tracks analysis

A speed filter was applied on locations to delete speed data higher than 10 km · h^−1^, which is the maximum travel speed previously recorded by Macaroni penguins [41]. The temporal resolution of the oceanographic data limited us to subsample the tracks at four points per day. Therefore, we chose to keep locations closest to 04:00 h, 10:00 h, 16:00 h and 22:00 h (local time) which provided a 24 h cycle divided into 4 × 6 h periods.

It has been shown that penguins decrease their horizontal movements when increasing their foraging activity, especially during the central phase of their trip [3, 57]. Thus, trips were split into three phases according to the smoothed relation between the heading velocity and the elapsed time relative to the departure. First, the outward phase, indicating the journey between the island and the central phase, was defined as the initial contiguous period where the smoothed heading velocities were higher than the average heading velocity during the whole trip (2.2 ± 1.4 km · h^−1^, Table 1). Second, the central phase was defined as the period where the heading velocities were below the mean heading velocity. Finally, the inward phase, from the central phase to the colony, corresponded to an increase of the heading velocity. In addition, as Macaroni penguins forage less at night [13, 58], we excluded from the analyses the velocities between 22:00 and 03:00 which, respectively, correspond to local dusk and dawn [59]. Directions of penguins and currents they experienced (varying from 0 to 360°) were then computed for each location using the Great Circle distance (*bearing* function, “geosphere” package).

The distribution of sub-mesoscale structures were investigated in two ways. Firstly, we looked at “the distribution of eddies within each trip phase” computed as $\frac{n_{locs- eddies\ }\ in\kern0.5em trip\ phase\ }{n_{locs- eddies}\ on\kern0.5em the\ whole\ trip\kern0.5em } \times 100$ for each trip phase respectively. *n*_*locs* − *eddies*_ indicates the number of locations within an eddy. Secondly, we looked at the “degree of association with eddies” computed as $\frac{n_{locs- eddies\ }\kern0.5em }{n_{locs\ }\ in\ trip\ phase\kern0.5em } \times 100$, for each trip phase respectively. Same ratios were computed for investigating filaments distributions.

### Statistical analysis

All analyses were conducted in the R statistical environment [60]. We used circular statistics (“circular” package) to determine the average bearing of currents and animals within each phase and assess the correlations between currents and animal directions. A Mann–Whitney *U* test (“stats” package) was used to compare the behavior of penguins within and outside of eddies or filaments. Following these descriptive analyses, different linear mixed effects models (*lme* function, “nlme” package) were constructed. For all models, individuals were included as a random effect as each location within individuals was not independent. The autocorrelation of residuals was tested (*acf* function) and consequently an autoregressive term of order 1 (coAR1) was included.

The best model was selected using the Akaike criterion (AIC [61]). Firstly, to investigate the response behavior to occurrence of eddies within each trip phase, three mixed models (one by trip phase, called M1, M2, M3) were built with the heading velocity as response variable and the factor “occurrence of eddies” (explanatory binary variable: absence or presence). Secondly, another model was built to link the variation of heading speed to the occurrence of frontal structures (explanatory binary variable: absence or presence). This model (not presented) had an AIC higher than the null model and the weak number of filaments within the outward and inward phases prevented us from building one model per trip phase. Thus, we looked at the relation of heading velocity (response variable) with the FSLE values (explanatory variable) when penguins were inside filaments (FSLE >0.1, model called M4). Finally, we tested the influence of currents (explanatory variable) on the travelling speed (response variable, model called M5). The different studied parameters are presented as Mean ± Standard Deviation (SD) whereas coefficients of models are presented as Mean ± SE (Standard Error). AIC deviations of tested models from the null models are shown. Results were considered significant at *P* < 0.05.
